# IBS-Symptoms in IBD Patients—Manifestation of Concomitant or Different Entities

**DOI:** 10.3390/jcm10010031

**Published:** 2020-12-24

**Authors:** Patrycja Szałwińska, Jakub Włodarczyk, Antonino Spinelli, Jakub Fichna, Marcin Włodarczyk

**Affiliations:** 1 Department of Biochemistry, Medical University of Lodz, Mazowiecka 6/8, 92-215 Lodz, Poland; patrycja.szalwinska@stud.umed.lodz.pl (P.S.); jakub.wlodarczyk@umed.lodz.pl (J.W.); jakub.fichna@umed.lodz.pl (J.F.); 2 Department of General and Colorectal Surgery, Medical University of Lodz, Haller Square 1, 90-624 Lodz, Poland; 3 Colon and Rectal Surgery Division, Humanitas Clinical and Research Center IRCCS, Rozzano, 20089 Milano, Italy; antonino.spinelli@hunimed.eu; 4 Department of Biomedical Sciences, Humanitas University, Pieve Emanuele, 20090 Milano, Italy

**Keywords:** irritable bowel syndrome, inflammatory bowel diseases, irritable bowel symptoms, remission, fecal calprotectin, inflammation, gut–brain axis

## Abstract

Irritable bowel syndrome (IBS) is a functional heterogenous disease with a multifactorial pathogenesis. It is characterized by abdominal pain, discomfort, and alteration in gut motility. The occurrence of similar symptoms was observed in patients in clinical remission of inflammatory bowel diseases (IBD) that is Crohn’s disease (CD) and ulcerative colitis (UC), which pathogenesis is also not fully understood. Hence, arose the question if these symptoms are “true IBS” imposed on IBD, or is it a subclinical form of IBD or even pre-IBD? In this article, based on a narrative overview of the literature, we try to find an answer to this query by discussing the pathogenesis and overlaps between these conditions.

## 1. Introduction

Irritable bowel syndrome (IBS), sometimes also called “spastic colon”, is a heterogenous, chronic disease with multifactorial pathogenesis [1]. Depending on diagnostic criteria, evaluated population, access to health care, and cultural impact, the prevalence of IBS fluctuates between 10 and 25%. IBS affects mostly adults in Europe and North America, especially females, and generally manifests between the age of 15 and 65 years [2,3]. Alteration in gut motility (abnormal gut contractions resulting in irregular stool pattern: diarrhea in IBS-D, constipation in IBS-C, or both in IBS-M), visceral hypersensitivity (increased sensation of physiological stimuli and pain), and overactivity of the immune system are believed to play a fundamental role in IBS pathogenesis. Nevertheless, other factors, such as genetic predisposition, stress, anxiety, food intolerance, changes in the gut–brain axis, and gut microbiota, can also be connected with the genesis of this disorder [1,4,5]. Interestingly, one study [6] revealed that IBS is associated with a disproportionately high prevalence of abdominal and pelvic surgery, for example, cholecystectomy, appendectomy, and hysterectomy, in comparison to the general population. This connection between IBS and surgical procedures may be a consequence of imprecise pre-operative diagnoses (as IBS symptoms may overlap with other conditions treated by surgery) or, alternatively, a result of promoting IBS by these interventions [7]. However, the exact mechanisms supporting this hypothesis are not yet known.

Inflammatory bowel diseases (IBD) are a group of conditions that involve chronic inflammation of the digestive tract, represented mainly by Crohn’s disease (CD) and ulcerative colitis (UC). IBD affect mostly patients between the ages of 15 and 30 years; however, they may occur at any age and remain gender-neutral [2]. IBD have been considered a condition affecting highly developed countries, e.g., the US in North America and the UK in Europe, but nowadays, an increased prevalence of IBD in Asia, Oceania, and sub-Saharan Africa are also observed. That is probably caused by industrialization, improved socioeconomic status, as well as dietary and lifestyle changes [8].

The pathogenesis of IBD is not completely understood. However, changes in the enteric nervous system (ENS), such as an increase in the number of ganglion cells [9], axonal degeneration [10], infiltration of inflammatory cells [11], and presence of major histocompatibility complex class II molecules [12], have been reported and suggest that the hyperactivation of the immune system is associated with the development of IBD. What is more, according to a study by Roberts et al. [13], gut dysfunction in patients with active IBD may be connected with increased prostaglandin levels as a result of COX-2 overexpression in neural cells of the myenteric plexus. Another study revealed increased substance P and its receptor content in patients with CD [14,15] and a shift from mainly cholinergic to substance P-positive innervation in patients with UC [16]. Moreover, CD-positive patients present lower numbers of interstitial cells of Cajal (ICC)—mesenchymal cells of the gastrointestinal (GI) tract (a so-called “pacemaker system”), which are responsible for proper GI motility [17]. Concurrently, visceral hypersensitivity has also been reported to play a role in IBD pathogenesis. However, according to many studies, visceral hyperalgesia probably depends on the activity of the disease, type of predominant inflammation (acute or chronic), and occupied region of the GI tract [18]. Taking all these findings together, it seems that inflammation in IBD patients affects many parts of the neuromuscular apparatus and results in gut function changes. Moreover, dysfunction of the intestinal barrier combined with pathological immune reactions appears to be fundamental for IBD pathogenesis [3].

Drugs used in IBS, depending on the subtype, include antidepressants, antibiotics, peripherally restricted opioids, as well as more specific or experimental ones, such as serotonin receptor ligands, CFTR (cystic fibrosis transmembrane conductance regulator) channel antagonists, GC-C (guanylate cyclase C), and CIC-2 (type 2 chloride ion channels) activators [3]. Treatment of IBD involves anti-inflammatory and immunomodulatory drugs, such as corticosteroids, derivatives of 5-aminosalicylic acid, thiopurines, azathioprine, and more, with an individual approach to every patient taking specific factors into consideration (for example, activity, course, duration of the disease, response to previous medications, extra-intestinal manifestations) with the aim of achievement and maintenance of steroid-free remission (clinically and endoscopically defined). It should be emphasized that in IBD, the association between symptoms and lesions is poor, so the therapy should be based on structural changes revealed in endoscopic examination (treat to target). In both disorders, modification of lifestyle and dietary habits is an important part of the management plan.

### Methods

The materials for this narrative review were searched for in the following databases: PubMed, Embase (OVIDversion), and Google Scholar. The search query consisted of the combination of the following keywords: “inflammatory bowel disease”, “irritable bowel disease”, “irritable bowel syndrome”, “post-inflammatory bowel disease syndrome”, “gut–brain axis”, “irritable bowel symptoms”, “Crohn disease”, and “Ulcerative colitis”. Results were limited to relevant papers published in English. There were no restrictions for the publication date for the articles cited in all subsections of the manuscript. The first search was performed on 11 February 2020, updated on 3 September 2020, with a final revision on 4 December 2020. References in all the included studies were reviewed for more eligible articles. Each article was reviewed independently by three (PSz, JW, and MW) researchers for inclusion according to prior established inclusion and exclusion criteria. Disagreements on article selection were resolved through discussion until consensus was reached or resolved by discussion with JF and AS. Conference abstracts were excluded. Articles were excluded in the case of non-English language, inaccessibility of the full text, preclinical research, and commentaries. Extracted details included study population and demographics, details of interventions and controls, study methodology, and information to assess bias.

## 2. IBS and IBD—Similarities and Differences

IBS and IBD seem to be quite separate entities, but still, they do share some similarities [2]. First, their symptoms overlap to some extent: They both may include abdominal pain, bloating, diarrhea, and watery stools, which can make it difficult to distinguish between these disorders. However, pain in IBS results from tension in the intestinal wall and can be relieved by defecation [19], while in IBD, it is more constant, and it may result from inflammatory cytokines impacting on afferent nerve firing [20]. Moreover, in the case of IBD, there are so-called “alarm symptoms”, such as fever, weight and appetite loss, bloody stool, vomiting, or anemia, which are absent in IBS [2]. Second, despite the fact that extracolonic symptoms may appear in the course of both diseases, in IBS, they are more general and include, for example, nausea or dyspepsia, while they seem to be more serious and disabling in IBD—they may affect joints, eyes, skin or liver. Furthermore, the epidemiology is slightly different—IBS may occur at any age and is seen more often in women, while IBD appear mainly in young adults between 15 and 30 years old and remain gender-neutral—as mentioned earlier. Phenotypic differences are also clear—in IBS, visibly normal mucosa is observed. On the contrary, in IBD, inflammation, ulcerations, fibrosis, and structuring can be seen during colonoscopy with the naked eye [21]. The pathogenesis of IBS and IBD is not completely understood; however, it is believed to be multifactorial. In both cases, it may include not only environmental and psychological factors (such as stress, depression, negative life events) but also genetic factors, enduring submucosal inflammation, and other changes involving the gut–brain axis and alteration in gut microbiota. Similarities and differences between those entities are summarized in Table 1.

### 2.1. Immune System Activation and Increased Permeability

The immune system plays a crucial role in the pathophysiology of IBS and IBD, regardless of the triggering factors [2]. In IBS, the recruitment of immune cells, for example, mast cells (the most common histological finding, responsible for the release of histamine, proteases, and chemokines) and lymphocytes, is observed. That also promotes local edema and increased levels of cytokines, such as IL-6, TNF, IL-1-β, which are currently identified to have a possible relationship with IBS and are also highly linked with depression and anxiety, suggesting the role of the gut in proper brain functioning [1,22,23]. Higher serum levels of IL-6, IL-8, and TNF-α revealed in IBS suggest a role of systemic inflammation in this disorder [24,25]. Moreover, low-grade inflammation reported in IBS can stimulate visceral nerves and induce dysmotility—the most typical symptom of IBS [26]. Interestingly, postinfectious IBS (PI-IBS, a subtype which is a consequence of past history or history of non-recognized bacterial, viral, or parasite GI infections) is believed to be associated with inflammation and mucosal damage rather than sporadic IBS, and according to Sadeghi et al., its characteristic features are increased macrophages, T lymphocytes, and serum IL-6 [27]. On the other hand, IBD also involves the recruitment of immune cells—mainly lymphocytes, which also release proinflammatory cytokines, such as TNF, IL-23, IL-17A, and IFN-γ [3]. Moreover, the inflammatory environment in IBD promotes the recruitment of monocytes in the immature proinflammatory state, which are believed to intensify chronic inflammation in the gut [28,29]. The best-known immune pathway connected with IBD is the nuclear factor kappa-light-chain enhancer of activated B cells (NF-κB) [30]. It is responsible for hyper-activation in epithelial or immune cells in IBD patients and increased production of other cytokines: IL-6, IL-12, IL-23, IL-1-β, and TNF-α [30,31]. What is more, a study by Langer et al. revealed that the inhibition of IFN-γ (another upregulated cytokine linked with immune modulation in IBD) alleviates experimentally induced colitis in IFN-γ knockout mice [32]. Of note, IL-6 and IL-8, which are elevated in IBD, have also been reported to be increased in IBS, as mentioned above [33,34]. What is more, calprotectin and human beta-defensin 2 (markers of innate immunity activation) can fluctuate in both IBS and IBD: Calprotectin, used mainly for detecting IBD, can also be marginally elevated in IBS patients, and human beta-defensin 2 may be increased even equally to the levels seen in active UC [35,36]. In consequence, despite the fact that each disease engages different elements of the immune system, they share a common consequence—mucosal immune activation and increased intestinal barrier permeability, which may underlie the pathogenesis of both entities [2]. A study by Vivinus-Nebot M. et al. [37] revealed that mucosal permeability in IBD patients with IBS-like symptoms is clearly elevated in comparison to healthy controls and IBD patients without IBS manifestations. Moreover, this abnormality can open the way for other factors to influence the gut, such as bacterial and food antigens [20].

### 2.2. Alteration in Gut Microbiota

The intestinal microbiota are responsible for gut development, digestion, metabolism, proper development of the humoral and cellular mucosal immune system, and protection against pathogens [38,39]. Any changes in bacterial number and composition may result in dysregulation between host and microbes known as dysbiosis, which may be triggered by pathogens, inflammatory mediators, or any initiators that can provoke a reaction of the immune system (such as diet, medical treatment, gut transit, redox potential in the gut lumen) [40] and lead to the inappropriate functioning of microbiota [1]. Several studies confirm gut dysbiosis in both IBS and IBS, which may be secondary due to mentioned triggers or may primarily contribute to IBD symptoms themselves [20,40]. However, the results reported are somewhat ambiguous. There is a consensus, based on a meta-analysis of CD-positive patients, that Bacteroidetes and Enterobacteriaceae are highly increased, while Firmicutes are decreased in these patients in comparison to healthy controls [41]. On the other hand, growth of Bacteroidetes was also observed in PI-IBS [42]. At the same time, according to another study [43], the microbiota of patients with CD differ noticeably from IBS patients, especially in the number of Faecalibacterium prausnitzii (F, frequently decreased in CD) and mucosa-associated Escherichia coli (E, often increased in this condition). This study revealed that CD can be distinguished from UC and IBS by a decreased F:E ratio. What is more, according to Porter et al., intercurrent infectious gastroenteritis (IGE, foodborne illnesses caused mainly by Salmonella spp., Campylobacter spp., and others) may increase IBD risk in IBS patients, suggesting the existence of a complex interaction between these disorders [44]. Interestingly, there are some data about the role of gut microbiota in the regulation and modulation of the immune system in IBD. For example, according to Grainger et al., microbiota are needed for the production of IL-10, which downregulates IFN-γ responses, and according to Longman et al., for proper intestinal barrier repair through innate production of IL-22 [45,46]. Moreover, one study in mice showed that T-reg cells (which are decreased in IBD) contribute to gut homeostasis and are induced by the microbiota [47]. In general, all these features underline the complex relationship between IBS, IBD, microbiota, and the immune system and the necessity for further investigations in this area.

### 2.3. Genetic Factors

Recently, investigations on genetic predispositions to IBS and IBD gained more and more interest because of strong evidence that both diseases aggregate in families. One study [48] showed lower concordance of IBS in dizygotic twins and higher in monozygotic twins, suggesting the role of genetics. Another meta-analysis revealed that serotonin transporters (SERT) insertion/deletion was associated with IBS in Asians and Caucasians but only for those with IBS-C [49]. Apart from this, mutations in an ion channel gene TRPM8 and sucraseisomaltase or single nucleotide polymorphisms were found to also play a role in IBS pathogenesis, but this issue needs further research [50,51,52]. Simultaneously, the number of loci responsible for IBD vulnerability continually increases—NOD2, IRGM, ATG16L, and IL23R genes (responsible for detection and response to gut bacteria) can be an example [20].

Of note, there is a possible genetic link between IBS and IBD, i.e., polymorphisms in the TNFSF15 gene (a member of the TNF family, in charge of interferon production)—a risk factor of CD in Europeans and Asians also found in IBS [53,54,55,56]. This can support the theory that excessive immune stimulation plays a role in both IBS and IBD pathogenesis [20]. However, the same study indicated no association between TNFSF15 and 30 other IBD genes, suggesting that IBD occurrence requires other less common factors that are not required for IBS [54].

Summarizing, genetics is highly believed to be linked with the genesis of both entities, and the results of performed investigations are promising. However, there is a long way to the discovery of genes specific for these disorders, mostly due to the small sizes of the samples, lack of reproducibility in large data sets, and variability of the clinical phenotype, which makes many studies easily underpowered [1,20].

### 2.4. Gut–Brain Axis

The gut–brain axis is a bidirectional communication system linking the central nervous system (CNS) and the enteric nervous system (ENS) in the intestinal wall, which allows proper GI function (food intake, digestion, adequate bowel movements) [3,57]. Dysregulation of this system due to psychological stressors and emotional responses is believed to be one of the main causes of GI disorders, including IBS [58]. One study [59] proved that 72% of IBS patients had elevated anxiety, 36% depression, and 66% somatization levels. What is more, some studies revealed that anxiety can predict IBS development—this can be proof for its etiological role [60,61]. Experimentally induced anxiety via maternal deprivation or crowding stress in rats may lead to visceral hypersensitivity, increased mast cell count, and gut permeability, which are known as features of IBS [62,63]. Less evidence has been gathered on the role of the gut–brain axis in IBD pathogenesis. However, there are data pointing out that long-lasting stress increases the probability of a relapse in UC [64,65]. Apart from that, animal models of acute stress and anxious phenotype (induced by water avoidance or maternal deprivation) had highly activated innate immunity (also seen in human studies with stressors like academic exams) and reported exacerbation of spontaneous colitis, especially in genetically predisposed mice with IL-2-knockout [66,67,68]. The last one appeared to be connected with mast cells, mainly associated with IBS [20].

As mentioned earlier, psychological factors are considered to be involved in the pathogenesis of both disorders, but it seems that they are more crucial in IBS than in IBD [69]. IBS patients seem to be characterized by higher psychological distress and illness anxiety than patients with IBD, but IBS and active IBD patients have the same anxiety and depression levels. Nevertheless, an important role seems to be played by symptoms activity: Patients with IBS and IBD show little differences in psychological distress or psychological risk factors.

Psychotherapy and behavioral therapy are a part of IBS management as they have a confirmed beneficial effect on unpleasant symptoms. On the contrary, there are limited attempts of psychological interventions in IBD, but so far, they do not show signs of improvement in colitis itself, although they improve the quality of life in both CD and UC [70].

The autonomic nervous system (ANS, consisting of the parasympathetic, sympathetic, and enteric nervous system) also participates in intestinal function control, and its dysregulation is increasingly believed to play a role in initiating and perpetuating IBD; hence its neurons terminate in the gut wall, including the muscularis, mucosal epithelium, enteroendocrine cells, and can be sensitive to chemical mediators of inflammation [71]. Moreover, inflammatory signals from IBD intestinal mucosa can influence peripheral neuronal signaling, resulting in peripheral sensitization with the response of proinflammatory cells. Generally speaking, changes in ANS ganglia and nerve cell bodies’ morphology, with modified expression of neurotransmitters, may be responsible for altered communication between ANS and effector intestine cells. ANS imbalance may also play a part in the pathogenesis of IBS, but available data are not clear cut. The results of the ANS investigations vary between the studies and suggest that depending on the IBS subtype parasympathetic/sympathetic, tone, and outflow can be increased or decreased [72]. No morphological changes have been found so far. However, one study [73] confirms that the presence of chronic low-grade inflammation in IBS may influence and change vagal reflexes. Moreover, in another study [74], IBS patients had increased heart rate, which may be an indirect proof for cardiac sympathovagal imbalance. Nevertheless, the role of ANS in IBS pathogenesis requires better study. Changes in ENS will be discussed below.

### 2.5. Changes in Enteric Nerves

As mentioned earlier, some changes in ENS have been reported in both IBS and IBD. Analysis involving IBS showed that TRPV-1-immunoreactive fibers and mast cells were related to a higher abdominal pain score, suggesting their role in visceral hypersensitivity [75]. Whereas, regarding IBD, there are interesting data in this area, especially from the patients suffering from UC. One study showed [76,77] that the more severe UC is, the more substance-P-positive nerves are found in colonic biopsy samples. Increases were also found in nerves co-expressing substance P and TRPV1 nerves with a decrease in somatostatic nerves. As far as TRPV1 nerves are concerned, a research by Akbar A. et al. [78] revealed a strong correlation between the number of TRPV1-positive fibers and pain, suggesting its pain-mediating role. The occurrence of such changes in both IBS and IBD may be proof for common pathways in the pathophysiology of these conditions and, further, a future direction for common pain treatment [20].

## 3. Remission of IBD and IBS-Like Symptoms

Typical manifestations of IBS include abdominal pain, discomfort, and altered bowel habits from diarrhea to constipation and are time-varying. In recent years similar symptoms were observed in IBD patients who were in clinical remission, and their appearance may be problematic for both patients and doctors, since, for the former, it may be a considerable source of stress and impairment of life quality, while the latter may find it difficult to distinguish between IBD relapse or IBS manifestations. [2]. Hence, the question of whether these symptoms are “true IBS” imposed on IBD or is it a subclinical form of IBD or even pre-IBD despite diagnosed remission by suitable criteria arose.

Some data about the prevalence of this phenomenon have already been gathered. The most recent meta-analysis by Faibrass et al. [79] revealed that 28.7% of patients in UC remission and 36.6% in CD remission reported IBS-like symptoms—substantially, this phenomenon was more common in those with CD than UC. Altogether, 35.2% (ranging from 11.2% to 63.6%) of patients diagnosed with IBD, in general, have symptoms similar to IBS without ongoing disease activity. This range comes from different remission criteria used in particular studies from the mentioned systematic review and severe heterogeneity between the studies, for example, different data collection or poor use of objective measures of remission (such as endoscopy or fecal sampling). What is more, according to Simren et al. [80], IBS-like symptoms appear to correspond rather with the duration of the disease than with patients’ age, administered treatment, or extension of the disorder. However, they were more frequent in females, especially with high levels of anxiety and depression [1,2,79,81].

Remission of IBD can be successfully achieved by proper treatment, as mentioned earlier [3]. This temporary reduction in severity of the disease is stated not only by the absence of symptoms and lower activity of the disease but also by endoscopic and laboratory findings.

Endoscopy evaluates, among others, the presence of macroscopic inflammation, which may be manifested by edema, erythema, bleeding, erosions, or ulcerations, and is carried out based on specific scales, for example, Mayo or Matts’ endoscopic score [82]. However, according to the latest findings, there are some discrepancies between the results of endoscopic and histopathological examinations, and the absence of mucosal inflammation does not exclude its presence in deeper layers of the gut, and that could be the reason for its improper function in both IBS and IBD. Consequently, there is evidence that some populations of IBS patients without endoscopic changes still can have histological abnormalities pointing to low-grade inflammation, such as infiltration of gut mucosa with several inflammatory cells, for instance, T cells (CD3+, CD4+, CD8+), macrophages, B cells, and mast cells, which may be responsible for the presence of symptoms [83]. These findings were originally reported in patients with postinfectious IBS (PI-IBS)—a subtype that develops in previously healthy individuals usually after bacterial or viral GI infection and possibly overlapping mostly with IBD [20,83]. Low-grade mucosal inflammation can also be found in UC patients even with clinical and endoscopic remission, which may be associated with the development of IBS-like symptoms [83]. However, a study by Henriksen et al. [84], in which patients 20 years after diagnosis of UC were included, revealed that inflammation does not seem to influence the prevalence of IBS-like symptoms, and its occurrence does not differ between individuals with and without ongoing inflammation (based on histological findings from colonic biopsies) [85]. Summarizing, although there is no clear and direct association between endoscopic and histological remission and prevalence of IBS-like symptoms, mostly due to the limited number of investigations, low-grade mucosal inflammation can be observed in both entities. Nevertheless, its influence on the prevalence of IBS-like symptoms in IBD patients remains unknown and still needs further research [83,84].

Fecal calprotectin (FC) level is used by many clinicians as a laboratory indicator of GI disorders. Calprotectin is a calcium- and zinc-binding protein released mainly by neutrophils—immune cells that migrate to the GI tract as a result of existing inflammation and increased permeability of gut mucosa—and is considered to be a positive acute-phase protein [86]. Since its amounts correspond well with intestinal inflammation and can reflect mucosal healing, calprotectin found its use in IBD diagnosis, monitoring the activity of the disease, treatment management, and prediction of relapse or remission [86]. What is more, it is used in practice to distinguish IBD from IBS. However, studies about FC as an objective marker for these disorders are not conclusive. In a study by Keohane et al. [87] (2010), 38.6% of patients in UC remission suffered from IBS-like symptoms (diagnosed on the basis of Rome II criteria) and had significantly elevated FC levels. Another research by Jonefjall et al. [88] (2016), in which remission was defined by endoscopic findings and FC level (<200 μg/g), found that only 18.2% of patients reported such symptoms. That speaks for the theory that low FC levels with proved endoscopic remission can be connected with reduced incidence of IBS-like symptoms [83]. Interestingly, recent studies using Rome III criteria were opposite to this thesis, saying there was no correlation between FC levels and presence of IBS-like symptoms and revealed no statistical difference between calprotectin levels of patients in clinical remission with and without IBS symptoms, although they were higher in the clinically active group [83,84,87,88,89,90,91]. Differences in the conclusions of studies may arise from dissimilar criteria defining remission and IBS symptoms, as well as study population, sample size, or quantity [83]. It is worth mentioning that one study [91] revealed that in some groups of patients with IBS manifestations, FC levels were slightly elevated (100–200), which testifies for low-grade inflammation that may be responsible for these incidences. What is more, 31% of patients reporting IBS-like symptoms had normal FC levels. However, an FC level below 40 μg/g in patients reporting IBS-like symptoms has been shown to be connected with a 1% chance of IBD, which makes it a useful biomarker in directing patients on screening colonoscopy [92]. Interestingly, the prevalence of IBS symptoms in IBD was higher when the FC level <100 μg/g was used to define remission and lower when an endoscopic or histological assessment was taken into consideration [79]. Overall, it is worth remembering that basing diagnosis on fecal calprotectin remains debatable since it lacks a reference standard and a cut-off value clearly defining remission and also is not specific for IBD—it may play a role in the management of infectious gastroenteritis, acute appendicitis, celiac disease, or even cystic fibrosis [86].

Currently, because IBS-like symptoms occur more and more often in IBD patients, there is a need for guidelines that will make it easier for doctors to take better care of such patients. The AGA Clinical Practice Update may be an example [93]. This expert review presents best practices in the diagnosis and management of GI symptoms in patients with IBD, which may be useful in everyday routines, such as the exclusion of the ongoing inflammatory process (by measurement of FC levels, endoscopic examination with biopsy, cross-sectional imaging), exclusion of anatomic abnormalities (which may cause obstructive symptoms like constipation, nausea, vomiting, abdominal pain), FC monitoring, low FODMAP diet, psychological therapies, probiotics, and laxatives, in patients with chronic constipation. Importantly, overlapping functional GI symptoms in IBD patients should be taken into consideration when they are persistent, and the ongoing inflammation has been excluded. Experts suggest ruling out disorders such as chronic pancreatitis, bile acid diarrhea, SIBO, and enhanced visceral sensitivity. Diagnosis of IBS-like symptoms should involve anorectal manometry (to rule out pelvic floor disorders) and the balloon expulsion test in those with defecatory disorders (e.g., chronic constipation, fecal incontinence, overflow diarrhea) as these conditions may respond to biofeedback therapy [94]. The Practice Update also underlines the role of brain–gut axis abnormalities as a potential pathophysiological cause of such symptoms, which can be treated by physiological therapy or antidepressants, provided this diagnosis is properly evaluated. Regarding the management of patients with IBS-like symptoms in patients with quiescent IBD, the efficiency of the low FODMAP diet has been confirmed in a randomized control study [95]. Cox et al. stated that a 4-week low FODMAP diet is safe and helpful in managing persistent gut symptoms. However, as mentioned above, some issues require further investigations, which may contribute to the development of a specific management plan for IBS-IBD patients and ensure the best possible treatment.

## 4. True IBS or Subclinical IBD?

The occurrence of symptoms similar to IBS in patients in clinical remission of IBD is not an infrequent phenomenon and is a kind of challenge for both health care workers and patients. However, it is still unclear if this should be interpreted as true IBS imposed on IBD or as a manifestation of IBD with continuing subclinical inflammation despite the presence of remission [2]. Apart from the issues mentioned earlier, there are other convincing arguments for both hypotheses, and they will be presented below.

The argumentation for the presence of “true IBS” coincidental with IBD is persuasive and convinces that these entities can coexist. First, it is statistically possible that some IBD patients can develop IBS de novo during clinical remission of IBD due to the high prevalence of IBS in the general population [2]. Second, the IBD-IBS population can be clearly distinguished from asymptomatic IBD patients in remission based on the Rome III or II criteria for IBS. Apart from that, IBS-like symptoms in IBD remission meet the most typical features of true IBS on both a general (they are more common in females and are positively correlated with anxiety—a well-known trigger of IBS) and molecular level (in these patients, increased intestinal permeability, a higher number of TRPV1 nerve fibers and lower expression of tight junction proteins zonulin-1 and alpha catenin—generally typical for IBS—were observed) [2,78,96]. Besides, a study by Aguas et al. [97] revealed that IBS is more frequent in first-degree relatives of patients with IBD, which may suggest shared genetic vulnerability and the possibility that these patients can predispose to IBS development next to IBD. There is also a possibility that there are some unknown shared genetic-environmental risk factors crucial for both diseases resulting in the coincidence of IBS and IBD [2].

However, these arguments do not convince everyone. First, the Rome criteria for IBS underline the fact that there should be no organic explanation for the symptoms (abdominal pain, bloating, diarrhea, constipation), so maybe they should not be used to define IBS in individuals already diagnosed with IBD regardless of disease activity [2]. What is more, alteration in gut permeability is common for IBS and IBD and cannot be used to distinguish these two disorders. Moreover, both of them involve improper activity of the immune system, but one could argue that the inflammatory process in IBD cannot be compared to gentle, variable, and sometimes even absent inflammation in IBS, suggesting that they might be different entities [2,37]. A study by Vivinus Nebot et al. [37] revealed increased levels of the proinflammatory cytokine TNF and a higher count of intraepithelial lymphocytes in remission of IBD with IBS-like symptoms, which is common in IBD but absent in IBS. Finally, as mentioned above, fecal calprotectin levels may remain normal despite the occurrence of IBS symptoms in IBD patients in remission, suggesting that not all IBS-like symptoms in IBD can be explained by ongoing inflammation [87,91,98].

## 5. Conclusions

Summarizing, IBS and IBD share many common symptoms, and there are some overlapping mechanisms in these disorders, such as increased gut permeability, altered immune system activation, inflammation, or changes in ENS and gut microbiota. Patients in IBD remission complaining of IBS-like symptoms still pose a diagnostic and therapeutic dilemma. Nevertheless, attempts to classify these symptoms as “true IBS” or subclinical IBD are insufficient as they do not account for all available observations. Hence, more investigations in this area are needed since they may lead to a consensus on this issue and provide a suitable and most effective therapy for these individuals [2].

## Figures and Tables

**Table 1:** Similarities and differences between irritable bowel syndrome (IBS) and inflammatory bowel diseases (IBD).

	Irritable Bowel Syndrome	Inflammatory Bowel Diseases
**Pathogenesis**	Multifactorial. Common mechanisms: increased gut permeability, altered immune activation, inflammation, changes in ENS and gut–brain axis, changes in gut microbiota, psychological factors (stress, depression).	
**Epidemiology**	At any age. More common in females.	Mainly young adults (15–30 y.o.) Gender-neutral.
**Symptoms**	Abdominal pain, discomfort, bloating, diarrhea, watery stools	
**“Alarm symptoms”**	Absent	Fever, weight and appetite loss, bloody stool, vomiting, anemia
**Pain**	Resulting from tension in the intestinal wall. Can be relieved by defecation.	Probably resulting from the impact of inflammatory cytokines on afferent nerve firing. More constant, cannot be relieved by defecation.
**Extracolonic symptoms**	For example, nausea, dyspepsia	Affecting joints, eyes, skin, liver. More serious and disabling.
**Endoscopic lesions**	Absent.	Present. Inflammation, ulcerations, fibrosis, structuring.
**Treatment**	Antidepressants, antibiotics, opioids, serotonin receptor ligands, CFTR channel antagonists, GC-C, and CIC-2 activators. Psychotherapy and behavioral therapy.	Anti-inflammatory and immunomodulatory drugs.
	Modification of lifestyle and dietary habits in both.	

Abbreivations: ENS (enteric nervous system), CFTR (cystic fibrosis transmembrane conductance regulator), GC-C (guanylate cyclase C), CIC-2 (type 2 chloride ion channels).

## Data Availability

No new data were created or analyzed in this study. Data sharing is not applicable to this article.
